# Endoplasmic Reticulum-Targeted Subunit Toxins Provide a New Approach to Rescue Misfolded Mutant Proteins and Revert Cell Models of Genetic Diseases

**DOI:** 10.1371/journal.pone.0166948

**Published:** 2016-12-09

**Authors:** Humaira Adnan, Zhenbo Zhang, Hyun-Joo Park, Chetankumar Tailor, Clare Che, Mustafa Kamani, George Spitalny, Beth Binnington, Clifford Lingwood

**Affiliations:** 1 Division of Molecular Structure and Function, The Hospital for Sick Children, Toronto, Ontario, Canada; 2 Division of Cell Biology, Research Institute, The Hospital for Sick Children, Toronto, Ontario, Canada; 3 ERAD Therapeutics, Caledon, Ontario, Canada; 4 Department of Biochemistry, University of Toronto, Ontario, Canada; 5 Department of Laboratory Medicine & Pathobiology, University of Toronto, Ontario, Canada; Institut Curie, FRANCE

## Abstract

Many germ line diseases stem from a relatively minor disturbance in mutant protein endoplasmic reticulum (ER) 3D assembly. Chaperones are recruited which, on failure to correct folding, sort the mutant for retrotranslocation and cytosolic proteasomal degradation (ER-associated degradation-ERAD), to initiate/exacerbate deficiency-disease symptoms. Several bacterial (and plant) subunit toxins, retrograde transport to the ER after initial cell surface receptor binding/internalization. The A subunit has evolved to mimic a misfolded protein and hijack the ERAD membrane translocon (dislocon), to effect cytosolic access and cytopathology. We show such toxins compete for ERAD to rescue endogenous misfolded proteins. Cholera toxin or verotoxin (Shiga toxin) containing genetically inactivated (± an N-terminal polyleucine tail) A subunit can, within 2–4 hrs, temporarily increase F508delCFTR protein, the major cystic fibrosis (CF) mutant (5-10x), F508delCFTR Golgi maturation (<10x), cell surface expression (20x) and chloride transport (2x) in F508del CFTR transfected cells and patient-derived F508delCFTR bronchiolar epithelia, without apparent cytopathology. These toxoids also increase glucocerobrosidase (GCC) in N370SGCC Gaucher Disease fibroblasts (3x), another ERAD–exacerbated misfiling disease. We identify a new, potentially benign approach to the treatment of certain genetic protein misfolding diseases.

## Introduction

Endoplasmic reticulum associated degradation (ERAD) is a cellular quality control mechanism by which the three dimensional folding of nascent polypeptides is sampled for aberrant features [1]. Proteins deemed to have achieved a suboptimal three-dimensional structure by interplay with a variety of ER-located chaperones, are targeted for cytosolic transfer, ubiquitination and proteosomal degradation. This homeostatic pathway ensures that only fully functional proteins are allowed to traffic to their functional sites within the cell. ERAD is linked to several pleotropic responses in the cell, e.g., autophagy [2] and the unfolded protein response [3], to provide latitude to accommodate ER stress which may occur during protein metabolic fluctuations.

Many human genetic diseases (>30) originate from mutations which result in a minor misfolding of the mutant protein [4], such that significant function is retained in in vitro assay. In such diseases, ERAD can precipitate or exacerbate deficiency disease symptoms. Thus, development of methods to rescue such mutant proteins from ERAD have become a focus for disease treatment strategies. Several approaches have been used to attempt to correct or bypass the misfolding of the mutant protein, for example, pharmacological chaperones[5–8], proteosomal inhibitors [9,10] or inhibitors of ERAD components[11,12] to decrease degradation.

The mechanism by which misfolded proteins within the ER are translocated to the cytosol for degradation is complex. The nature of the translocon (or dislocon [13]) is as yet, incompletely defined. Members of the Derlin protein family are central and although the role of reverse transit of the Sec61 translocon in ERAD has become contentious[14,15], the Sec61 translocon [16–18] may yet be involved.

This translocon is selectively hijacked by the A subunit of various plant and bacterial protein subunit toxins, which require cytosolic access for A subunit induction of cellular damage [19–21]. This pathway also intersects the cytosolic transit of antigenic peptides for immune recognition [22,23]. Within the ER lumen, the C terminus of these A subunits mimics an unfolded/misfolded protein and co-opts the ERAD chaperones for cytosolic egress[24,25]. Proteins involved in ERAD translocation are also involved in toxin A subunit cytosolic transfer[21,26,27]. Once internalized into cells[28], these toxins undergo receptor mediated retrograde transport, from endosomes, to the trans-Golgi network, Golgi and finally target the endoplasmic reticulum[29,30]. Here, the subunits separate and the furin-clipped A subunit[31] is translocated into the cytosol. The A subunits have evolved to avoid proteosomal cleavage via restriction of the lysine content required for ubiquitination[24].

The fact that toxin A subunit and ERAD substrates utilize the same/similar translocon machinery for ER-cytosolic egress and that theoretically, only one protein can occupy the translocon at a time, suggested that such toxoids could provide a new, general, competitive means to temporarily reduce the transit of endogenous ERAD substrates into the cytosol for degradation. Since the A subunit is a translocon substrate, any ERAD inhibition would be temporary and lost once translocated. This provides impetus to study the potential efficacy of toxoid rescue of ERAD substrates.

We now show this to be the case for verotoxin (VT, Shiga toxin) and primarily, cholera toxin (CT). The pentameric B subunit of these toxins bind different glycosphingolipid receptors (Gb_3_, globotriaosyl ceramide and GM1, monosialo gangliotetraosyl ceramide, respectively) as a means to achieve ER access, and thereby target a different cell subset. GM1 is present in most human cells and therefore most cells are CT sensitive, whereas Gb_3_ expression is restricted to epithelial/endothelial cell subsets and VT cell sensitivity is therefore far more restricted[32]. Both receptors mediate toxin retrograde ER transport [33]. VT A subunit is an RNA glycanase which blocks protein synthesis[34], while CTA is an ADP-ribosyl transferase which activates adenyl cyclase[35].

We have used the inactivated A subunit containing holotoxoids [26, 27], to show the efficacy to reduce ERAD substrate degradation in cell culture models of F508delCFTR, and N370S GCC Gaucher disease. Addition of an N-terminal polyleucine hydrophobic (stop-transfer) [36] sequence to increase translocon residence time, shows the potential to enhance this approach to develop highly bioactive vectors for the targeted, rapid, temporary blockade of ERAD and potential benign amelioration of any ERAD-based disease. These toxoids were without overt cell culture cytopathology (apoptosis).

Given their distinct mechanism of action, these toxoids may be able to complement other misfolded protein rescue strategies [37,38].

## Materials and Methods

A-subunit inactivated bacterial holotoxoids were generated by site specific mutagenesis as described for by Wen *et al* [39] for VT1 and Douce *et al* [40] for CT. Toxoids were generated with an N terminal 6 His tag and purified by Ni column affinity chromatography. A subunit N terminal polyleucine extension holotoxoids were generated by overlap PCR as described in the SI file. CT0 was also generated from an *E*.*coli* codon optimized plasmid without His tag and purified by galactose agarose affinity chromatography [41].

### Cell culture and treatment

Human embryonic kidney (HEK 293T) cells stably transfected for overexpression of wt CFTR or F508delCFTR [42] and the homozygous F508delCFTR patient derived bronchial epithelial cell line CFBE [43] were respectively, kindly supplied by Drs Rotin and Bear at this hospital,. Cells were cultured in DMEM supplemented with 10% (v/v) FBS, 0.1mM non essential amino acids, selective antibiotics, gentamycin and G418, 5 mg/ml in 5% CO2 at 37°C. Gaucher patient derived N370S GCC skin fibroblasts [44] were grown in MEM with 10% (v/v) FBS with 5% penicillin and streptomycin. Cells were mycoplasma free. In experiments 1 x 10^6^ cells were plated in 12 well dishes and grown for 18h at 37°C before treatment. Cells were treated with toxoid for different incubation times at 37°C.

### SDS-PAGE and western blot analysis

Cells were harvested with lysis buffer (TNTE) plus protease inhibitor complete mini and three freeze/thaws were performed. The cell extracts were sonicated on ice for 10 min and centrifuged at 9000 × g for 20 min at 4°C. Protein was quantified by BCA assay. Protein (20μg) was separated on 6% SDS-PAGE, transferred to nitrocellulose membranes, then blocked in 5% milk in Tris-buffered saline with 0.1% Tween 20 and incubated overnight with mouse monoclonal anti CFTR (1:1000) antibody (CF3 Mab anti CFTR (Abcam)) or rabbit anti glucocerobrosidase (Sigma). After 1 h of incubation with either horseradish peroxidase-conjugated anti-rabbit or anti-mouse secondary antibodies, bands were detected by chemiluminescence (ECL Plus) and visualized using a LiCOR, Odyssey ® Fc Imaging system. The densitometric analysis of protein was determined using Image J (NIH software). Vinculin or calnexin expression were immunodetected as a protein loading control. In some cases, Ponceau protein stain of the gel served as a loading control.

### Immunofluorescence

Cells grown on glass coverslips were treated as indicated then fixed with 3% paraformaldehyde/PBS. Cells were permeabilized with 1% methanol prior to treatment with M3A7 Mab anti CFTR (Santa Cruz 0and FITC-WGA. Cell bound antibodies were detected with Alexa488 or Alexa594-conjugated goat anti- mouse antibodies. Images were obtained using an Olympus IX81 inverted fluorescence microscope (60Å~oil immersion, NA 1.35) equipped with a Hamamatsu C9100-13 backthinned EM-CCD camera and Yokogawa CSU X1 spinning disk confocal scan head. Image acquisition, deconvolution and cropping were performed with Volocity software. Composite images were assembled using Adobe Photoshop.Experiment was repeated 4x.

### Cl¯ ion transport fluorescence polarization FLIPR assay

Quadruplicated cultures of cells expressing F508del CFTR were grown at 37°C until 5 days postconfluency in 96 well plates, followed by incubation with toxoids for 4 h at either 27°C or 37°C. FLIPRTETRA [45], which can measure plasma membrane polarization as a function of chloride transport [46], was added for 45 min after removing media from cells and plate was read on a fluorescent plate reader. Results were compares to corrector VX809 treated cell cultures.

### Assay of primary F508del CFTR bronchiole epithelial monolayers chloride transport

Third party Ussing chamber assay of primary F508del CFTR epithelia were carried by out by Charles River Discovery and are permitted under in-house Ethics Approval Chloride transport function of triplicate CFhBE F508delCFTR monolayers grown in Snapwell™ filter inserts was monitored at 37°C as the CFTR agonist evoked short circuit current output of an Ussing epithelial voltage clamp apparatus. The short circuit current was assayed in response to VX770 potentiator after channel activation with forskolin and compared to that induced by corrector VX809 or CT0 toxoid.

### GCC assay

Glucocerebrosidase activity was measured in cell lysates using methyl umbelliferyl glucose as artificial substrate in citrate/phosphate buffer [44].

## Results

### Functionally active Verotoxin rescues F508del CFTR

**Fig 1:**
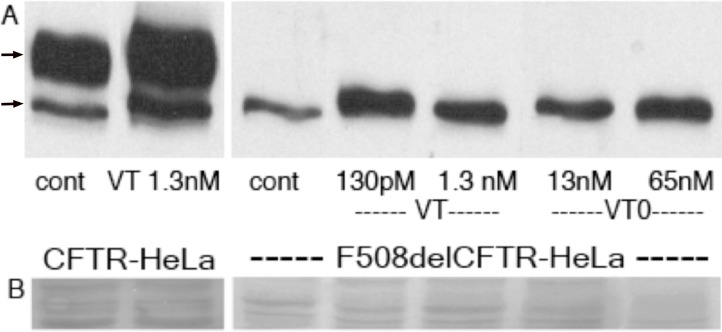
VT increases cellular CFTR and F508del CFTR. HeLa cells transfected with wild type CFTR or F508del CFTR (were grown for 2hrs ± VT or A subunit inactive VT (VT0). Cells were solubilized in SDS, separated by PAGE and subject to western blot using anti CFTR; arrows = mature band c(upper) and immature band b (A). Ponceau S protein stain of the CFTR molecular weight range provides a loading control(B).

Wildtype VT (130pM) was found to increase F508del CFTR, the major mutant responsible for cystic fibrosis[47], the most common ERAD-dependent genetic disease, in transfected (Gb_3_ expressing) HeLa cells within 2hrs (Fig 1), even though protein synthesis is completely shut down under such conditions[48]. CFTR separates as an immature (high mannose carbohydrate) band b and mature (complex oligosaccharide) band c, by western blot [49]. The intensity of band b is reduced and band c is largely undetectable for F508del CFTR as compared to wild-type CFTR expressing cells. In our results, band b accumulated by ~5 fold after VT treatment but no band c was detected in these HeLa cells. Low dose VT was more effective. Wildtype CFTR is also subject to ERAD[50] and was also ‘rescued’ by VT1 treatment (both bands b and c) of transfected HeLa cells. VT0 (inactivated VT) was also effective to rescue F508del CFTR in these cells.

### A-subunit inactive CT holotoxins rescue F508del CFTR

**Fig 2:**
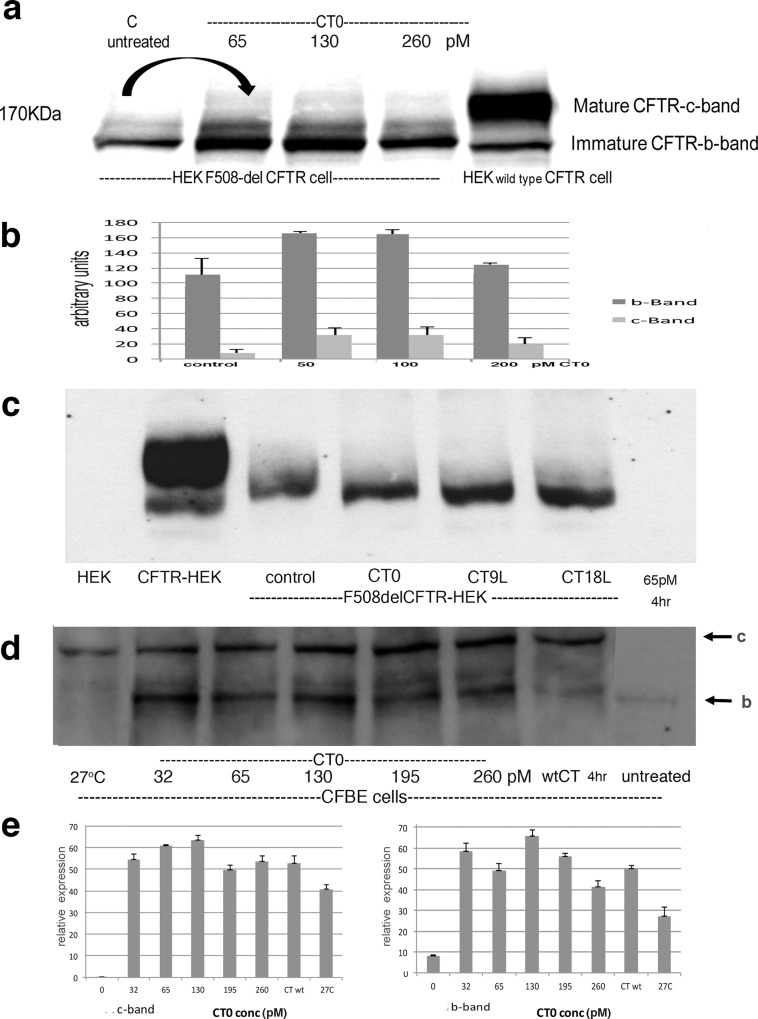
Catalytically inactive CT0 increases F508del CFTR Western blots were stained with anti CFTR. **a**)HEK cells transfected with F508delCFTR were incubated for 4hrs ± 65- 260pM A subunit inactivated CT0; the arrow emphasizes the comparison of untreated baseline to maximal CT0 enhanced CFTR expression, **b**) quantitation of F508del CFTR rescue, **c**) F508delCFTR HEK cells were treated with 65pM CT0 containing a 9(CT9L) or 18(CT18L) leucine N-terminal A subunit extension for 4 hr, d) CFBE cell line was treated with increasing CT0 for 4hr and compared to cells rescued by 27°C culture-arrows = band c (upper) and band b, e) Quantitation of F508delCFTR increase in CFBE cells -relative scale ±SEM. Groups were compared using ANOVA followed by Tukey’s test [104].

Mutations which inactivate A subunit catalytic activity [39,40] were made in CT and VT1 (SI file). The benign, A subunit inactivated CT0 (0.65–2.60nM) was effective to rescue F508delCFTR from ERAD in HEK transfected cells (Fig 2A). Both b and c CFTR bands were rescued (Fig 2B).

Furthermore, a polyleucine N-terminal extension, corresponding to the minimal and maximal translocon stop transfer sequences [36], were added to potentially increase translocon residence time. (Sequencing of the plasmids confirmed the addition of 9L to VT0 and CT0 but 16L were added to VT and 18L to CT A subunits in the longer constructs- SI file). The efficacy of the inactive A CT0 toxoid for F508del CFTR rescue was increased by the polyleucine extensions (Fig 2C). Figure A in S1 File shows a dose response for CT18L. Toxin B subunit does not rescue F508delCFTR (Figure B in S1 File).

Treatment of the CFBE bronchiolar epithelial cell line, derived from a F508del CFTR patient [51], showed a similar CT0 dose response (Fig 2D). Both band b and band c showed major (20-50x) increase (Fig 2E). Culture of the cells at 27°C known to rescue the temperature dependent F508del CFTR protein [10] also induced a shift from band b to band c (~30x - Fig 2E), but, unlike the CT0 treated cells, there was little concomitant increase in band b.

### Rescued F508del CFTR can be detected at cell surface

**Fig 3:**
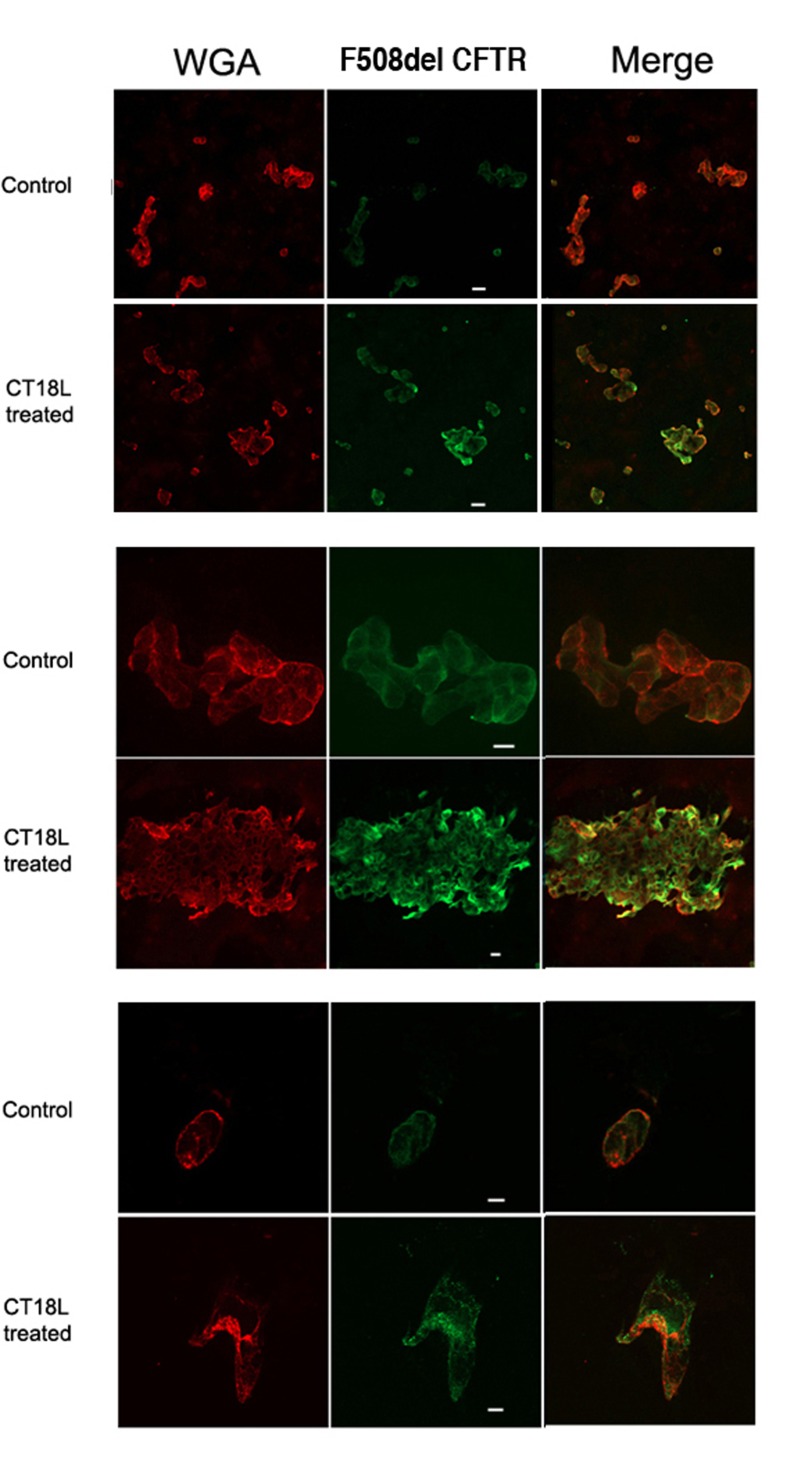
Cell surface expression of F508del CFTR rescued by CT18L F508delCFTR expressing HEK cells were treated with CT18lL (260pM) for 4hr at 37°C and compared to control untreated cells by double labeling with wheat germ agglutinin to label the cell surface, and MabM3A7 anti CFTR. Colabelling (yellow) was only seen for treated cells. This experiment was repeated four times. Bar = 20μm.

Some of the CT18L rescued F508delCFTR was expressed on the cell surface (Fig 3) as determined by colabelling with anti CFTR and a cell surface staining lectin, WGA. Untreated F508delCFTR -HEK cells were weakly stained for CFTR, if at all. The antiCFTR Mab M3A7 used binds to a cytosolic domain of CFTR and therefore cells required methanol permeabilization prior to immunostaining. Only by colocalization with WGA staining, can cell surface CFTR staining be determined. Significant levels of F508delCFTR are detected which do not colocalize with WGA, consistent with an increase in both intracellular (ER) and plasma membrane F508delCFTR.

### Cholera toxoids promotes F508delCFTR cell chloride transport

#### a) FLIPR assay of F508delCFTR expressing cell lines

**Fig 4:**
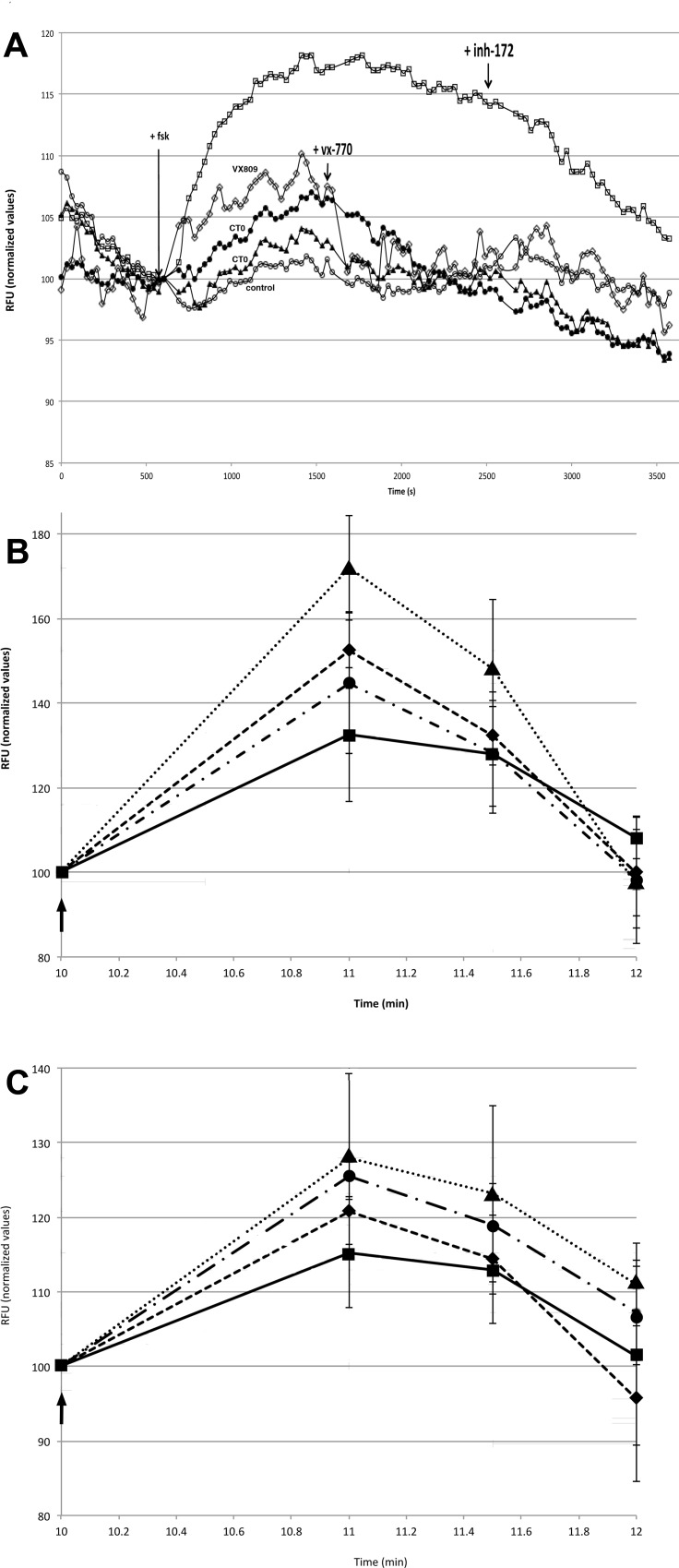
**Effect of CT0 on F508delCFTR-HEK and CFBE epithelial cell chloride transport measured by FLIPR assay A**) Fluorescence measurement (relative fluorescent units, chloride transport surrogate) of quadruplicate F508delCFTR-HEK cell cultures maintained at 27°C for 4hr,○-○ untreated control F508delCFTR cells, □-□ Wt CFTR cells, ◇-◇ 3μM VX809 treated F508delCFTR cells, ●-● 65pM CT0 treated F508delCFTR cells, ▲⋯▲ 260pM CT0 treated F508delCFTR cells. Mean of 4 replicate cultures is shown ±SD. 10μM Forskolin, 100nM VX770 activator and 20μM CFTR inhibitor 172 were added to all cultures at the time marked by the arrows. **B)** Quadruplicate CFBE cell cultures at 37°C ± 2hr treatment, ■___■untreated control F508delCFTR cells ●-∙-∙● 3μM VX809 treated F508delCFTR cells, ◆_ _ _◆130pM CT0 treated F508delCFTR cells, ▲⋯▲13pM CT0 treated F508delCFTR cells. Mean ±SD. **C)** Quadruplicate CFBE cell cultures at 37°C ± 4hr treatment, ■^**__**^■ untreated control, ●-∙-∙● 3μM VX809 treated cells, ◆_ _ _◆130pM CT0 treated cells, ▲⋯▲13pM CT0 treated cells. Mean ±SD. Arrows in B and C mark 10μM forskolin addition.

In order to address the potential clinical utility of this approach, the mutant CT0 was expressed from a plasmid adapted for *E*.*coli* codon usage and purified by affinity chromatography [41] by a third party (SI file). The effect of this CT0 (Figure C in S1 File) on membrane polarization of F508delCFTR HEK and CFBE cells was measured by FLIPR assay [45], as a surrogate for chloride transport[46] (Fig 4A). An increase in depolarization was observed after forskolin activation of F508delCFTR HEK cells treated with 65 or 260pM CTO for 4hrs at 27°C. This increase was 75% (for 65pM CT0) and 40% (for 260pM CT0) of the increase seen for 4hr treatment with 3μM corrector VX809 [52] (Fig 4A). VX809 increased the chloride transport of F508delCFTR cells to 50% that of wildtype CFTR overexpressing HEK cells. Subsequent addition of CFTR activator VX 770 reduced the VX809 induced increase, consistent with previous reports [53,54], but was less effective to reduce the CT0 induced increase.

In CFBE cells, CT0-increased, FLIPR-monitored, chloride transport was measured after 2hr and 4hr of treatment (Fig 4B and 4C). In this F508delCFTR patient derived bronchial epithelial cell line, under some conditions, CT0 was more effective to rescue chloride transport than VX809 corrector and the kinetics of CT0 rescue were faster. A >2 fold increase in forskolin activated transport was seen after 2hr following 13pM CT0 treatment, as compared to a 35% increase for 3μM VX809 (Fig 4B). After 4hr, the VX809 induced correction increased to 70% whereas the 13pM CT0 increase was reduced to 85% (Fig 4C). At both 2 and 4 hr CTO treatment, the lower 13pM dose was more effective. Western blot confirmed a dose dependent increase in F508delCFTR (bands b and c) following CT0 treatment of CFBE cells at 37°C (Fig 2D).

#### b) Assay of F508del CFTR patient bronchiolar epithelial chloride transport

**Fig 5:**
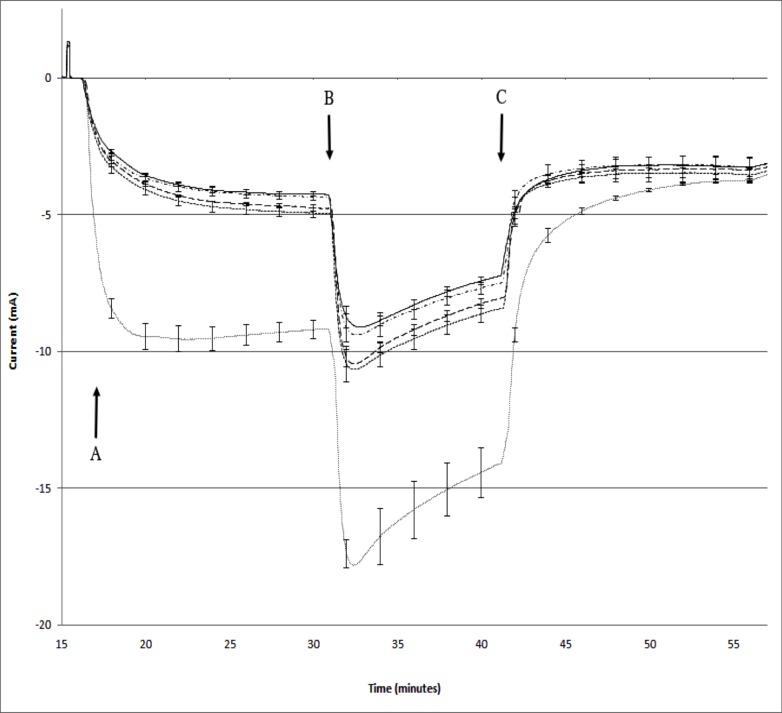
Effect of CTO on primary F508delCFTR bronchiolar epithelial cell monolayer chloride transport Triplicate F508delCFTR patient derived primary bronchiolar epithelial cell monolayers were treated with 13pM(-∙-∙-∙-∙), 130pM (^__ __ __^), 13nM(- - - -) CT0 or 3μM VX809(∙∙∙∙) and chloride channel function measured by short circuit current (±SEM) determined by an Ussing epithelial voltage clamp apparatus. CFTR was activated by 10μM forskolin addition (at A), augmented by addition of potentiator 0.1μM VX 770 (at B) and inhibited by 20μM CFTRinh-172 (at C). 13nM, 130pM, but not 13pM, CT0 induced a significant difference from vehicle at peak F508delCFTR mediated chloride transport: P>0.001 by Dunnett’s test [56]. CT0 achieved a maximum increase of 20% that observed for corrector VX809. There were no toxic effects at these concentrations of CT0 as indicated by normal, uniform transepithelial resistance measurements and uniform response to BaCl_2_ (not shown).

The galactose affinity purified CT0 was also monitored for efficacy of rescue of F058delCFTR plasma membrane chloride transport by a third party (Charles River Discovery) using Ussing chamber assay of primary patient-derived CFBE bronchial epithelia. Treatment of bronchiolar epithelial cell monolayers with CT0 at 130pM or 13nM for 4hr increased F058delCFTR chloride channel transport which was amplified by addition of potentiator VX770 and inhibited by inhibitor CFTR inh-172 (Fig 5). This increase was statistically significant using Student’s t-test [55] and by Dunnett’s test [56] (P<0.01) which is a multiple sample procedure for comparing several treatments with a control. 13pM CT0 treatment did not induce significant transport increase. The CT0 caused no toxicity and the increase was approximately 20% of that induced by corrector VX809 under similar conditions.

### Toxoids rescue N370S GCC in Gaucher cells

According to the mechanism of action we propose, whereby the toxoid A subunits compete for the ERAD translocon, our toxoid treatment regime should be effective in most diseases in which ERAD eliminates a misfolded, yet functional mutant protein. A further example of such a disease is the Gaucher glucosylceramide lysosomal storage disease.

The most frequent mutation in glucocerebrosidase (GCC) causing Gaucher disease, is N370S [57] which induces minor misfolding and ERAD elimination of GCC [58].

**Fig 6:**
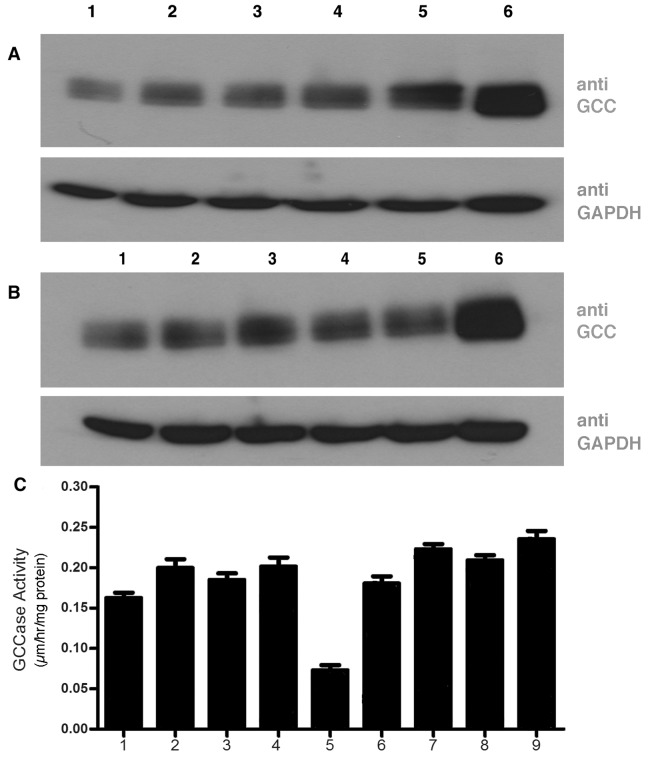
Efficacy of VT and CT constructs to rescue N370S glucocerebrosidase in Gaucher disease fibroblasts Primary skin fibroblasts from an N370S GCC Gaucher disease patient were cultured with ± 260pM toxoid constructs for 4hr and compared to WT fibroblasts. GCC content was determined by western blot (A,B) and enzyme activity(C): panels A (CT treated), B (VT treated): lanes 1- control cells, lanes 2- wt CT/VT, lanes 3- CT0/VT0, lanes 4- CT9L/ VT9L, lanes 5- CT18L/VT16L, lanes 6- normal fibroblast control. Lower panels in A,B = GAPDH western blot loading controls. panel C: GCC activity assayed in triplicate ±SD; lane 1 VT, lane 2-VT0, lane 3-VT9L, lane 4-VT16L, lane 5- untreated control, lane 6-CT, lane 7- CT0, lane 8-CT9L, lane 9-CT18.WT fibroblasts had an activity of 0.9μm/hr/mg protein.

A Gb_3_ expressing fibroblast cell line from a N370S type 1 Gaucher disease patient was selected to determine whether ERAD blockade using our toxoid constructs was a feasible means to rescue this GCC mutant to increase GCC activity and allow GCC traffic to lysosomes. The cell lysate of N370S mutant showed 3% GCC activity of normal fibroblasts. After toxoid construct treatment, an increase in N370S GCC could be detected by western blot, particularly for CT constructs (Fig 6). WT CT induced a 30% increase, similar to CT0(40%). CT09L induced 100% increase and CT18L increased GCC protein 3 fold. For VT0 constructs, rescue appeared less effective with WT VT giving a 10% GCC increase by western blot, VT0 30%, VT9L 20% and VT16L 25%. By western blot, the inactivated constructs were more effective compared to the intact holotoxins. However, when the enzyme activity of N370S GCC was measured, CT0 and VT0 constructs were equally effective to increase activity. Activity was increased to >300%, giving an activity ~20% that of wildtype cells. Thus, the level of GCC protein detected by western blot, may not fully reflect the functional rescue of the mutant protein.

## Discussion

The symptoms of many protein misfolding diseases are deemed to be precipitated or exacerbated by the ERAD quality control system. Cell models indicate a role in many more[59–68]. ERAD is a key component of homeostasis, and while several ERAD inhibitors have been developed [69] and tested for therapeutic (primarily antineoplastic) effect [70–72], they have to contend with the adverse effects of induction of ER stress [73] and the unfolded protein response (UPR)[74]. Accumulation of misfolded proteins can induce the UPR and lead to apoptosis[75]. Such effects can be used to advantage when using ERAD inhibitors against cancer [76,77], but become problematic in the treatment of protein misfolding diseases [9].

The use of ER targeted, exogenous, competitive substrates of the ERAD translocon provides a potentially benign means for the temporary down regulation of ERAD elimination of endogenous substrates, such as the partially misfolded mutant proteins involved in genetic disease. The method should be compatible with current use of pharmacological chaperones to correct protein folding [37,38]. The mutations made in the A subunit eliminated holotoxin cytotoxicity (>10^6^ fold reduction). Under standard conditions of protein rescue (1.3nM for 4hr), the toxoids we generated induced a UPR of ~2 (Figure D in SI file), far less than the thousand fold increase reported for other ERAD inhibitors [9]. A balance between ERAD inhibition to achieve mutant protein rescue and ER stress must be maintained [78] and this is promoted by the temporary nature of our ERAD blockade. It has been reported that ERAD blockade can decease ER stress [79] via an autophagy related mechanism.

We have shown that the Verotoxin, and more extensively, the cholera holotoxoids, are effective to partially rescue F508delCFTR and N370S GCC from degradation to increase their cellular function. Both wildtype VT and CT were also able to rescue F508delCFTR, despite potent protein synthesis inhibition by VT. B subunits of CT were not effective. The inactivated holotoxoids showed a dose dependent rescue of F508delCFTR, though the optimal dose varied for different cell lines, likely due to the relative toxin cell line sensitivity, which depends on many factors, including glycolipid receptor expression [80,81], its ceramide structure[82,83] and membrane organization [84,85, 86], A bell-shaped response may be due to additional B subunit signaling responses[87,88]. A dose of 100ng/mL (1.3nM) was generally effective. Band c F508delCFTR was increased 5–10 fold and chloride transport increased by up to 2 fold. CT0 induced responses were more rapid than those of corrector VX809(e.g. within 2hrs) but were transitory, consistent with the proposed reversible mechanism of action.

In Gaucher cells, CT0 treatment resulted in an N370S GCC activity which was 10% the normal GCC value. Published studies with pharmacological chaperones have achieved a similar N370S GCC increase [44]. Since our rescue is achieved by a different mechanism, these approaches are of potential synergy. However CTO increased activity was measured in the cell lysate. Thus, the effect on GCC maturation and lysosomal transit remain to be assessed.

The toxoid constructs we have generated will follow the normal B subunit mediated retrograde trafficking pathway of the parent holotoxins: after receptor mediated endocytosis–transit from endosomes to TGN to Golgi to ER [89]. The A subunit only separates from the B pentamer after reduction in the ER. This exposes the A subunit C terminal sequence which mimics a misfolded protein domain[24]. This ER targeting has been used as a means to deliver cargo e.g. anticancer drugs [90], peptides [22]. Our studies [91] and others [92] indicate the holotoxin itself is a potential antineoplastic.

The mechanism of A subunit (and ERAD substrate) translocation into the cytosol is still ill defined. How is the misfolded protein unfolded and what is the membrane translocation driving force? However, with the toxins, this problem is not confounded by nascent polypeptide ER anterograde import function. Co-precipitation with Sec61 should therefore, be considered less ambiguous. Toxin ER targeting has been assessed by Sec61 coprecipitation with ricin [93], cholera toxin [94] and verotoxin [95]. RNAi knockdown of Sec61 was shown to protect cells from verotoxin and Pseudomonas exotoxin but surprisingly, not ricin [96]. Ricin translocation was Derlin dependent [97,98]. Cholera toxin translocation is also Derlin dependent[99]. Our contention is that these toxins remain competitive substrates irrespective of the precise translocon composition.

The polyleucine tails we added are based on anterograde Sec61 transit. These increased efficacy of ERAD rescue, but may not be the most effective and certainly not translocon-specific sequences. However, the A subunit must remain a translocon substrate to ensure the reversible and temporary nature of the induced ERAD blockade.

Our experience has been that if cells are sensitive to VT, ERAD substrates expressed are rescued. Most cells express GM1 and are therefore CT sensitive. We observed no cell apoptosis for the toxoids (over 2 days) we have generated, even at 10^6^ fold higher dose than effective against ERAD.

Since CT targets most cell types, we focused on this toxoid. In F508delCFTR transfected HEK cells or CFBE cells, addition of 65-260pM CT0 for 4 hr is sufficient to induce a 5–10 fold increase in F508delCFTR band b and a corresponding increase in the lactosamine glycosylated (mature) band c F508delCFTR. As expected, CT B (or VT B subunits or heat inactivated VT) are without effect in this system. The significant increase in the mature F508delCFTR, band c, indicates some of the rescued protein in the ER, accesses the Golgi-located glycosylation machinery but the mechanism by which this is achieved is unknown. There are many impediments to F508del CFTR maturation[100] and intermediates other than bands c, b, may arise[101].

Using an antibody that binds a cytosolic domain of CFTR, we show that, after permeablization of cells cultured at 37°C, a major increase in cell surface F508delCFTR staining of CFBE cells is observed after toxoid treatment. F508delCFTR protein (bands b and c) are increased in the treated cells.

Using the FLIPR system[45,46], we find that 4h CT0 treatment of CFBE polarized bronchiolar epithelial cells from a F508delCFTR patient [51], induces a significant increase in plasma membrane chloride transport, greater, than that seen for 18hr treatment with corrector VX809.

CT0 expressed from an *E*.*coli* plasmid optimized for codon usage, showed a clear increase in CFBE and F508delCFTR patient derived primary lung epithelial cell chloride transport.

Temporary toxoid blockade of ERAD will result only in accumulation of the misfolded protein in the ER. The mechanism by which some of the increased ER protein escapes the ER and matures is unknown and was, for example, for F508del CFTR, quite variable. The opportunity for further cooperation with pharmacological chaperones is clear.

Although cell surface F508delCFTR is less stable than wildtype[102], multiple folding defects require correction[100] and mechanisms to potentiate the chloride transport function of plasma membrane F508delCFTR may also be necessary[103], our demonstration of a rapid, significant (but temporary) increase in functional F508delCFTR cell surface expression following reversible toxoid ERAD blockade, offers a completely new targeted mechanism to alleviate this, and potentially, other, ERAD-dependent protein misfolding diseases.

## Supporting Information

S1 File (DOCX)
