# Supplementary material for: Endoplasmic Reticulum-Targeted Subunit Toxins Provide a New Approach to Rescue Misfolded Mutant Proteins and Revert Cell Models of Genetic Diseases
Source: PLoS One. 2016 Dec 9;11(12):e0166948. doi: 10.1371/journal.pone.0166948 (PMC5147855; doi:10.1371/journal.pone.0166948)
Supplement: S1 File — (DOCX) [file pone.0166948.s001.docx]

**Supplementary Information**

**Construction of Mutated CT and VT**

**1. Cloning and inactivation of CT and VT**

CT was cloned from *Vibrio cholera* DNA provided by Ontario Public Health laboratory using CTFP/CTRP primer set and High-fidelity DNA polymerase (Invitrogen). PCR product was digested using EcoRI and BamHI, purified and inserted into linear pEGFP-N1 vector digested using the same restriction enzymes as the PCR product. The correct plasmid confirmed by sequencing, was then inactivated mutated at the locations S63K and P106S of CT A subunit ([1](#_ENREF_1)) by mutagenic primer sets MSKF/MSKR and MPSF/MPSR and Pfu High-fidelity DNA polymerase (Agilent Technologies) according to manufacturer’s guidelines. Sequences for all primer sets are displayed in Supplemental Table 1 and 2.

Inactivated VT with the mutations Y77S and E167Q ([2](#_ENREF_2)) was a generous gift from Dr. Alison O'Brien `s lab (Uniformed Services University of the Health Sciences, Bethesda, MD, USA).

**2. Addition of polyleucine to CTA and VTA subunit and sub-construction of expression plasmids**

The technique of overlap extension PCR was used to add 9 leucines (9L) and 18L for CT and 9L and 16L(2 L were lost in PCR) for VT between leader peptide and A subunit. Constructed plasmids (inactivated CT0, CT9L, CT18L and VT0, VT9L, VT16L VT) were sub-cloned into expression Vector pEcoli-Nterm 6xHN by using primer sets CTPF/CTRP for CT and VTPF/VTRP for VT.

After confirmed correct by sequencing, CT0, CT9L, CT18L and VT0, VT9L, VT16L were expressed in BL21 (DE3). The target proteins were purified using His-Tag purification kit according to manufacturer`s guideline (Qiagen).

**Supplemental Data**

**Table1: Primer Sequences for CT**

| Primers | Primer sequence |
| --- | --- |
| CTFP | 5`-ccggaattcATGGTAAAGATAATATTTGTGTTTT-3` (EcoRI) |
| CTRP | 5`-ccgggatcc CAAAACGGTTGCTTCTCATCATC-3` (BamHI) |
| MSKF | 5`-GATATGTTTCCACCAAAATTAGTTTGAG-3` |
| MSKR | 5`-CTCAAACTAATTTTGGTGGAAACATATC-3` |
| MPSF | 5`-TTAGGGGCATACAGTTCTCATCCAGATGA-3` |
| MPSR | 5`-TCATCTGGATGAGAACTGTATGCCCCTAA-3` |
| CT9LF | 5`-TTATCATCATTTTCATATGCACTGCTGCTGCTGCTGCTGCTGCTGCTG AATGATGATAAGTTATATCGG-3` |
| CT9LR | 5`-CCGATATAACTTATCATCATTCAGCAGCAGCAGCAGCAGCAGCAGCA  GTGCATATGAAAATGATGATAA-3 |
| CT18LF | 5`-TTATCATCATTTTCATATGCATTATTATTATTATTATTATTATTATTACTGCTGCTGCTGCTGCTGCTGCTGCTG AATGATGATAAGTTATATCGG -3` |
| CT18LR | 5`-CCGATATAACTTATCATCATTCAGCAGCAGCAGCAGCAGCAGCAGCAGTAATAATAATAATAATAATAATAATAATGCATATGAAAATGATGATAA -3` |
| CTPF | 5`-ccggtcgacATGGTAAAGATAATATTTGTGTTTT-3`(SalI) |
| CTPR | 5`-ccggaattcCAAAACGGTTGCTTCTCATCATC-3` (EcoRI) |

**Table2: Primer Sequences for VT**

| Primers | Primer sequence |
| --- | --- |
| VT9LF | 5`-TTTTCAGTTAATGTGGTGGCGCTGCTGCTGCTGCTGCTGCTGCTGCTG AAGGAATTTACCTTAGACTTC-3` |
| VT9LR | 5`-GAAGTCTAAGGTAAATTCCTTCAGCAGCAGCAGCAGCAGCAGCAGCAGCGCCACCACATTAACTGAAAA-3 |
| VT16LF | 5`-TTTTCAGTTAATGTGGTGGCGTTATTATTATTATTATTATTATTATTACTGCTGCTGCTGCTGCTGCTG AAGGAATTTACCTTAGACTTC-3` |
| VT16LR | 5`-GAAGTCTAAGGTAAATTCCTTCAGCAGCAGCAGCAGCAGCAGTAATAATAATAATAATAATAATAATAACGCCACCACATTAACTGAAAA -3 |
| VTPF | 5`-ccggtcgacATGAAAATAATTATTTTTAGAGTGC-3`(SalI) |
| VTPR | 5`-ccggaattcTCAACGAAAAATAACTTCGCTGAA-3` (EcoRI) |

The inactivated A subunit holotoxoids were purified based on their N terminal His CT0 produce using an *E coli* codon optimized plasmid without His tag was purified from the bacterial lysate by galactosyl-agarose affinity chromatography ([3](#_ENREF_3)). Bound holotoxin was eluted with 0.5M galactose.

**Figure A.** Dose response for CT18L rescue of F508delCFTR . Transfected HEK cells were treated with increasing doses of CT18L and subject to anti CFTR western blot. Anti CFTR western blot of F508delCFTR transfected HEK293 cells following 4hr treatment with increasing dose of CT18L.Lower panel shows immunoblot for calnexin as loading control. Right panels show quantitation of F508delCFTR band c and band b.


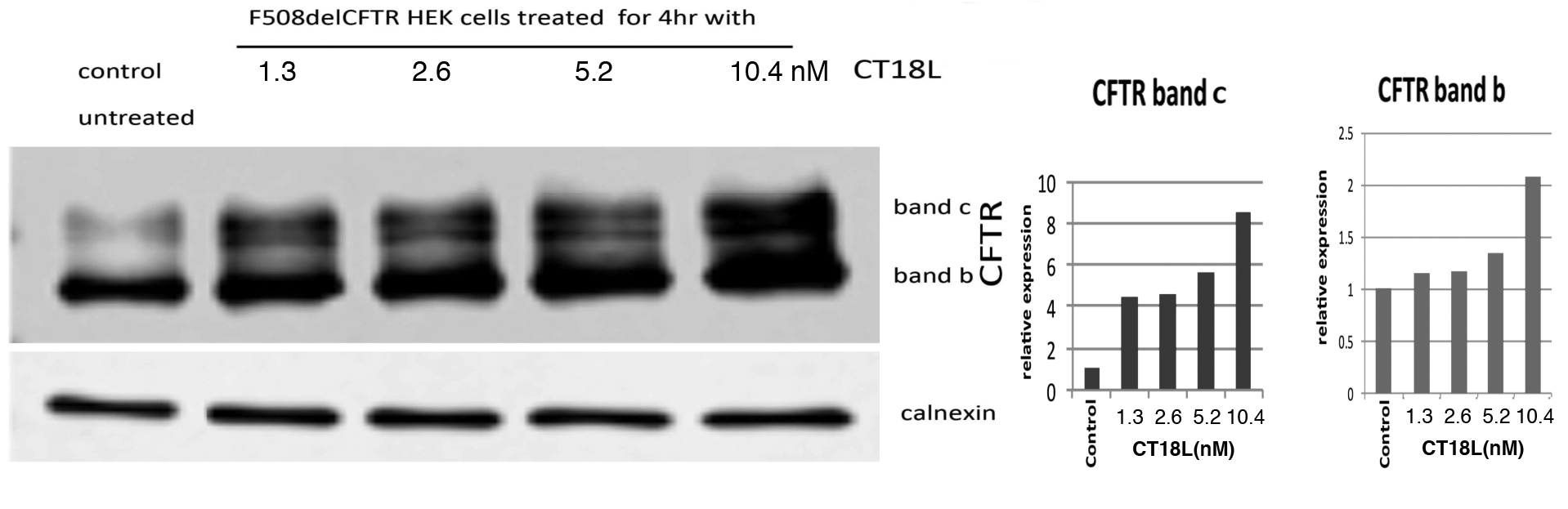


**Figure B.** F508delCFTR rescue requires toxoid A subunit. F508delCFTR-HEK cells were treated with increasing doses of CTB subunit or wildtype CT or CT18L for 4h and analysed by anti CFTR western blot. Immunostain of vinculin provided the loading control. Only wild type CT and CT18L treatment increased band b and band c. CTB did not affect CFTR expression


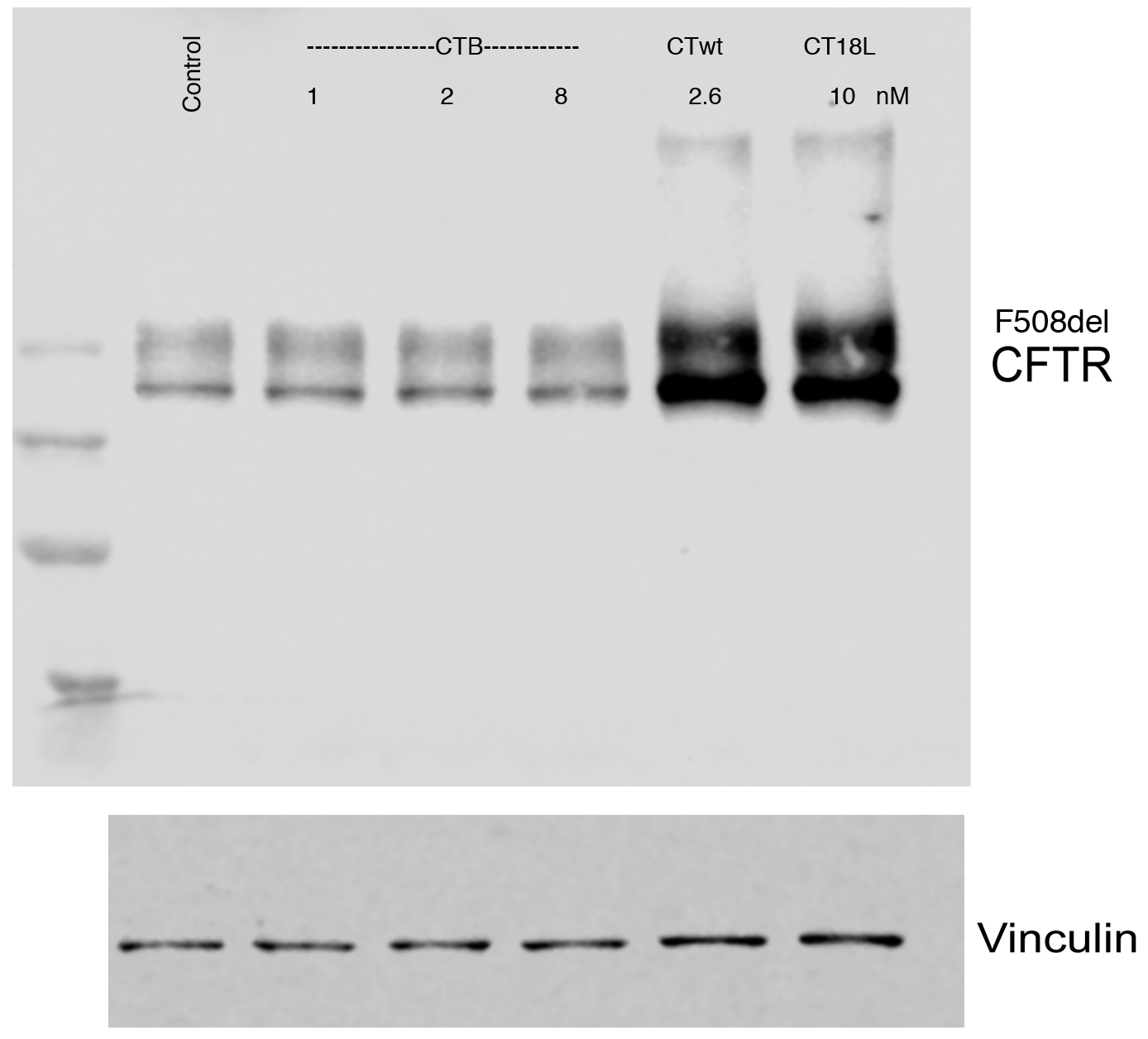


**Figure C.** Galactose affinity matrix purification of CT0. Panel A Coomassie blue protein stain of galactose eluate of galactosyl affinity matrix(3), panel B western blot with anti CT.

˙
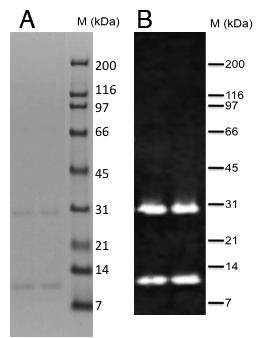


.

**Figure D**. Toxoids induce minimal UPR

F508delCFTR-HEK cells were treated with 130pM CT0, CT9L or CT18L for 4 hrs DNA was prepared. The Unfolded Protein Response was then measured by splicing of the transcription factor, XBP1 revealed by Pst1 digestion ([4](#_ENREF_4)). Accumulation of the 669bp fragment (boxed) is an index of UPR and is quantitated in the lower panel.


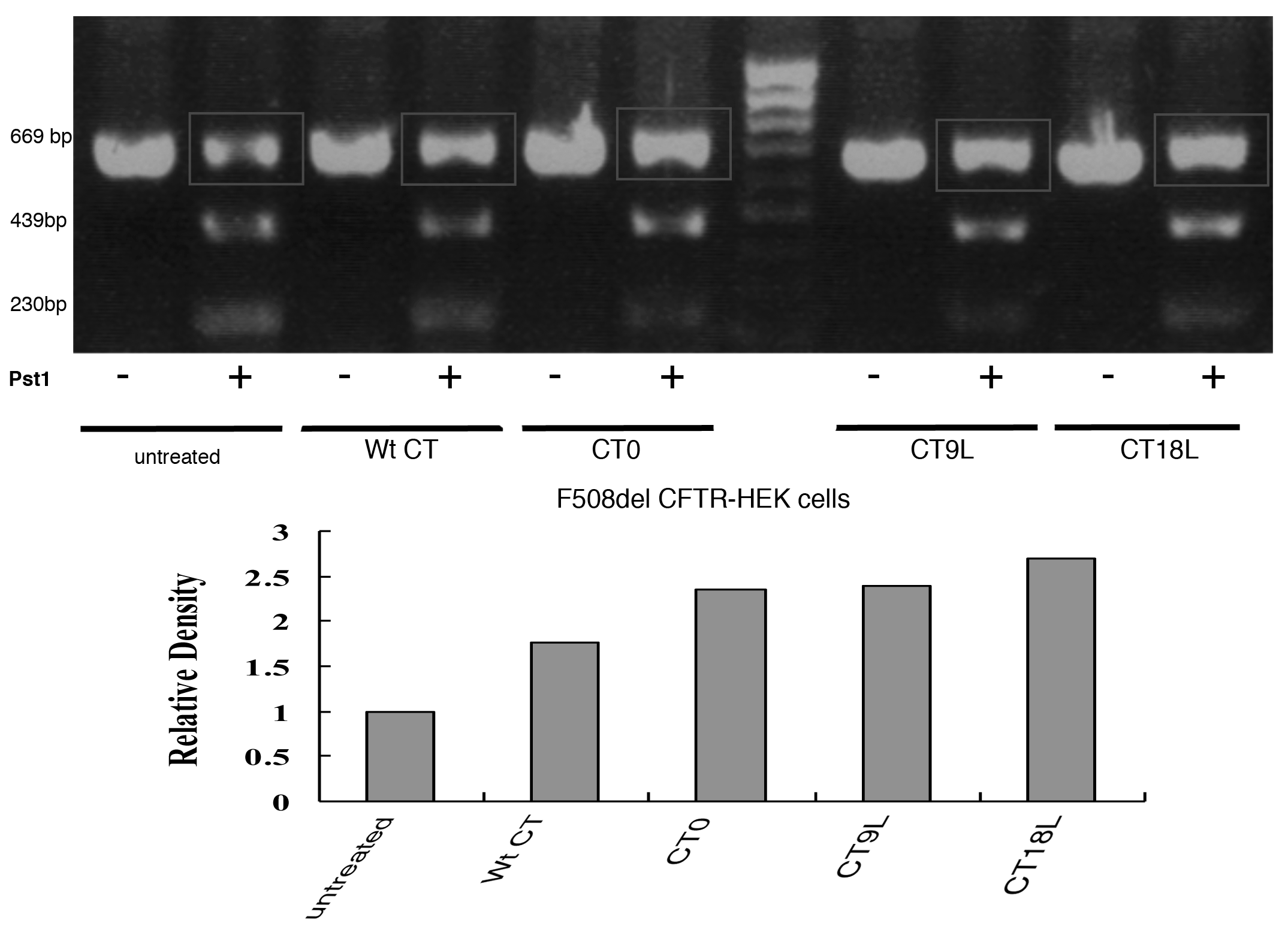


Supplementary References

1. Douce G, Fontana M, Pizza M, Rappuoli R, Dougan G. Intranasal immunogenicity and adjuvanticity of site-directed mutant derivatives of cholera toxin. Infection and immunity. 1997;65(7):2821-8. Epub 1997/07/01.

2. Wen SX, Teel LD, Judge NA, O'Brien AD. Genetic toxoids of Shiga toxin types 1 and 2 protect mice against homologous but not heterologous toxin challenge. Vaccine. 2006;24(8):1142-8.

3. Yasuda Y, Matano K, Asai T, Tochikubo K. Affinity purification of recombinant cholera toxin B subunit oligomer expressed in Bacillus brevis for potential human use as a mucosal adjuvant. FEMS Immunol Med Microbiol. 1998;20(4):311-8. Epub 1998/06/17.

4. Lemin AJ, Saleki K, van Lith M, Benham AM. Activation of the unfolded protein response and alternative splicing of ATF6alpha in HLA-B27 positive lymphocytes. FEBS letters. 2007;581(9):1819-24. Epub 2007/04/20.
